# Gas exchange measurements of carboxysome mutants reveal insights into cyanobacterial carbon-concentrating mechanisms

**DOI:** 10.1093/plphys/kiag221

**Published:** 2026-04-20

**Authors:** Rees Rillema, Anne Steensma, Berkley J Walker, Daniel C Ducat

**Affiliations:** Department of Biochemistry and Molecular Biology, Michigan State University, East Lansing, MI 48924, United States; MSU-DOE Plant Research Laboratory, Michigan State University, East Lansing, MI 48924, United States; MSU-DOE Plant Research Laboratory, Michigan State University, East Lansing, MI 48924, United States; Plant Biology Department, Michigan State University, East Lansing, MI 48924, United States; MSU-DOE Plant Research Laboratory, Michigan State University, East Lansing, MI 48924, United States; Plant Biology Department, Michigan State University, East Lansing, MI 48924, United States; Department of Biochemistry and Molecular Biology, Michigan State University, East Lansing, MI 48924, United States; MSU-DOE Plant Research Laboratory, Michigan State University, East Lansing, MI 48924, United States

## Abstract

Gas exchange measurements are widely used in plant research to quantitatively resolve photosynthetic performance and CO_2_ assimilation rates, yet gas exchange techniques are less commonly applied to algae and cyanobacteria. Cyanobacteria have a well-characterized carbon-concentrating mechanism (CCM) that includes a proteinaceous microcompartment called the carboxysome. Carboxysome structural components have been extensively studied and consist of a nucleated core of rubisco encapsulated by a diverse family of structurally related shell proteins. In addition to the dominant shell proteins that compose the majority of carboxysome facets, accessory shell components are widely distributed throughout cyanobacterial phyla. Herein, we utilize gas exchange techniques to elucidate the photosynthetic impacts of carboxysome mutants in *Synechoccocus elongatus* PCC 7942, emphasizing analysis of three accessory shell proteins: CcmK3, CcmK4, and CcmP. We detail technical considerations important for increasing the throughput and accuracy of gas exchange measurements using a recently developed commercial aquatic chamber. We find distinct photosynthetic phenotypes associated with the roles of different accessory shell proteins, with context-specific photosynthetic impairment in Δ*ccmP* mutants that contrast with the broader pleiotropic defects of Δ*ccmK3* Δ*ccmK4* strains under multiple environments. Our results have implications for understanding the role of accessory carboxysome shell components in facilitating carbon fixation and other carboxysomal functions.

## Teaser

Gas exchange techniques illuminate conditional photosynthetic deficiencies in mutants of the accessory shell components of the β-carboxysome.

## Introduction

**Figure 1:**
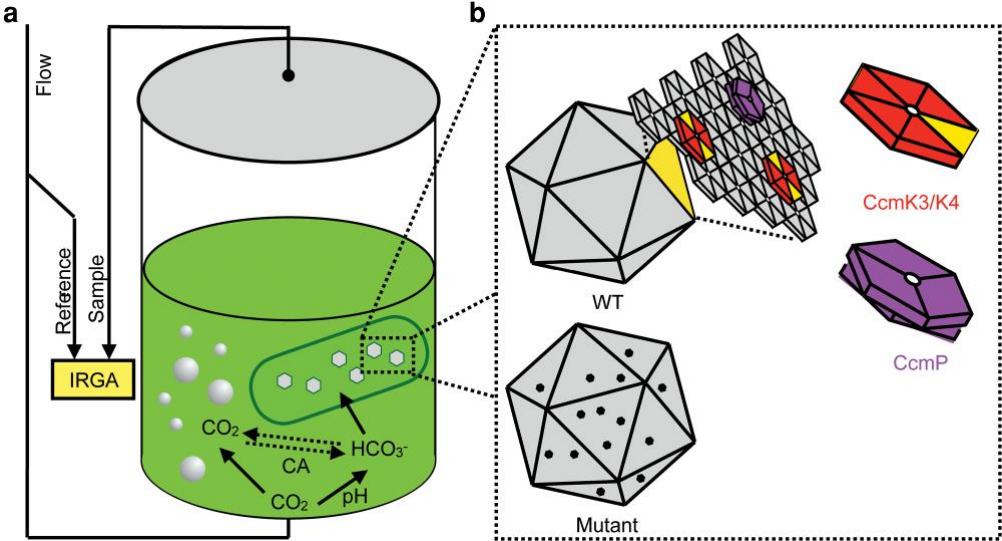
Schematic of liquid gas exchange chamber with carboxysome mutants. (a) Flow of mixed gas (solid arrows) enters the system and is bubbled into an aquatic chamber housing the cyanobacterial sample. Carbonic anhydrase (CA) accelerates the interconversion of dissolved CO_2_ with bicarbonate (dashed arrows). The infrared gas analyzer (IRGA) is used to compare the gas that has gone through a given sample. The difference between the reference and the sample is the portion assimilated by the cells. (b) Dashed box depicts a zoomed view of intracellular carboxysomes, including (top) WT carboxysome highlighting the minor shell CcmK3/K4 heterohexamers and CcmP trimer stacks and (bottom) a cartoon depiction of a mutant carboxysome lacking one or more of these shell components, which could be replaced by other shell proteins or potentially manifest as minor defects/gaps in the continuity of the shell surface.

Decades of development in both instrumentation and theory have firmly entrenched the use of infrared gas analyzers (IRGA) as a powerful approach for evaluating photosynthetic performance in plants, with implications across biological scales from molecular mechanisms to global ecological models (Long and Bernacchi 2003; Von Caemmerer 2013; Busch et al. 2024). Briefly, gas exchange is measured using IRGAs to quantify the difference in concentration between the flow of gaseous CO_2_ entering and leaving a given sample (Fig. 1a). Monitoring gas exchange in leaf tissues while systematically varying environmental properties (*eg*, CO_2_, light) provides a direct assessment of the core fluxes of the light and dark photosynthetic metabolic processes (Busch et al. 2024). For example, the net CO_2_ assimilation (A) response to intercellular CO_2_ concentration (Ci) is a foundational gas exchange measurement (*ie*, the A/C_i_ curve) routinely used to provide quantitative assessment of plant photosynthesis and physiology. Such A/C_i_ curves have been instrumental in building advanced models at all scales: from models of leaf- and plant-level physiology, to inform regional models of carbon cycling, and predictive models of the impacts of climate change (Farquhar et al. 1980; von Caemmerer 2000; Yin et al. 2021). While commercial IRGA-based systems for measuring gas exchange are well-developed for monitoring CO_2_ fluxes on leaf tissue, commercial instrumentation compatible with the analysis of liquid suspensions has only recently become available. Therefore, the use of infrared gas exchange techniques commonly used to assess plant photosynthesis have been less frequently applied to the evaluation of aquatic phototrophic microbes such as algae and cyanobacteria.

One primary technical barrier to the application of gas exchange techniques to algal and cyanobacterial cultures is the aqueous intercellular phase of planktonic cultures. Most notably, slow equilibrium rates between gaseous CO_2_ and dissolved HCO_3_^−^ in liquid samples limits the sampling rate and confound the interpretation of A/Ci measurements. These problems are exacerbated by the higher temperature and pH typically used to cultivate planktonic cyanobacteria and microalgae. Rohnke *et al* circumvented this difficulty by removing media and measuring gas exchange parameters of *Fremyella diplosiphon* in a “leaf-like” state on filter paper (Rohnke et al. 2020). However, this required rapid processing and careful attention to the moisture content of filtered cells to avert the loss of cell viability. Alternatively, researchers have utilized isotopic approaches to estimate gas exchange rates in planktonic cultures, such as approaches relying on the preferential use of ^13^CO_2_ over ^12^CO_2_ by ribulose 1,5-bisphosphate carboxylase/oxygenase (rubisco; Sharkey and Berry 1985; Hanson et al. 2014). Additionally, membrane-inlet mass spectrometry (MIMS) methods to discriminate between isotopes of O_2_ and CO_2_ have been adapted to liquid samples to evaluate microalgal and cyanobacterial gas exchange (Douchi et al. 2019; Santana-Sanchez et al. 2019; Burlacot et al. 2020). While isotopic approaches are increasingly used to analyze algae and cyanobacteria, they require customized and expensive equipment that can be technically challenging to operate and maintain. Together, these difficulties highlight the need for an accessible technique that uses common terminology of plant research while tailored to the unique characteristics of cyanobacterial cultures.

In addition to technical considerations, there are significant differences in the cellular structures and molecular mechanisms found in aquatic phototrophs relative to plants. Critically, all cyanobacteria have at least some form of a carbon-concentrating mechanism (CCM). Cyanobacterial CCMs include bicarbonate pumps that actively concentrate bicarbonate from the environment, and specialized compartments containing carbonic anhydrases (CAs) that catalyze rapid interconversion of HCO_3_^−^ ←→ CO_2_ (Badger and Price 2003; Burnap et al. 2015). These proteinaceous bacterial microcompartments are called carboxysomes, and encapsulate both CA and rubisco, so bicarbonate entering the carboxysome, is converted into CO_2_, enriching substrate availability near rubisco's active site. Carboxysome shells are composed of three classes of shell proteins: hexamers (BMC-H), trimers (BMC-T), and pentamers (BMC-P) (Kerfeld and Melnicki 2016). Most cyanobacterial genomes encode more than one copy of BMC-H and BMC-T genes, with one homolog acting as the most abundant, or major, shell protein (Sutter et al. 2021). For example, CcmK2 is the most abundant BMC-H protein in the assembled carboxysome shell in *Synechoccocus elongatus*, yet the genome contains additional hexamer subunits, CcmK3 and CcmK4 (Fig. 1b, top) (Rae et al. 2012). Likewise, *CcmO* is trimeric shell protein encoded in the core carboxysome operon of *S. elongatus* (Cai et al. 2013; Cameron et al. 2013), while *ccmP* encodes an additional BMC-T that is conserved throughout β-carboxysome containing bacteria. The function of “minor” shell proteins remains poorly understood, though it has been shown that they vary in their abundance depending on environmental conditions and therefore they are hypothesized to alter the permeability of the carboxysome shell (Cameron et al. 2013; Sommer et al. 2019; Sun et al. 2019; Sun et al. 2025). For example, a key distinction between CcmO and CcmP is that the latter protein is part of a distinct trimeric shell protein family proposed to form a “stacked” conformation, whereby a tightly oppressed dimer of the trimeric tandem domain is incorporated into the shell (Fig. 1b, bottom) (Cai et al. 2013; Larsson et al. 2017). Structural studies suggest that CcmP regulates the passage of larger metabolites, such as the phosphorylated substrate (*ie*, ribulose 1,5-bisphosphate) and products (*ie*, 3-phosphoglycerate) of rubisco, although this hypothesis lacks in vivo evidence (Melnicki et al. 2021; Sutter et al. 2021; Sun et al. 2025).

Herein, we utilized a recently developed LI-COR Aquatic Chamber (LAC) to collect gas exchange data for planktonic cultures of cyanobacteria (Hupp et al. 2021). We selected a range of mutants in carboxysome components in the model cyanobacterium, *S. elongatus*, to quantitatively evaluate the sensitivity of gas exchange techniques via the LAC. During these analyses we varied environmental conditions of light and CO_2_, as these have been previously shown to correlate to changes in carboxysome component abundance (Sommer et al. 2019; Sun et al. 2019; Sun et al. 2025). We present multiple technical and experimental considerations that may assist interested researchers in utilizing this instrumentation for cyanobacteria and other photosynthetic microbes. Our approach provides quantitative data on the contribution of minor shell components in modulating the efficiency of the cyanobacterial CCM which would be difficult to assess using standard metrics for microbial characterization.

## Results

### CO_2_ equilibration is rate limiting for aquatic chamber throughput

As a practical matter, adapting gas exchange techniques to aqueous samples requires protocol optimization to overcome the problem of CO_2_ exchange between the gaseous and aqueous phases. Gaseous CO_2_ must dissolve in water, disproportionate into bicarbonate, and equilibrate with the pool of existing inorganic carbon, parameters of which vary based on pH (Moroney et al. 2001; Talekar et al. 2022). While CO_2_ gas delivery can be modulated by LI-COR instrumentation on short time scales (*eg*, seconds), gas uptake/release for each data point of the carbon assimilation curve must be evaluated only once the system has reached steady state and the gas phase is fully equilibrated with the liquid growth medium. For example, when CO_2_ supplied to a liquid sample is increased, apparent rates of CO_2_ uptake are artificially inflated at early time points due to the gradual disproportionation of CO_2_ gas into bicarbonate in the void volume of the culture liquid. Device program settings restrict variation in the average slope and standard deviation of ΔH_2_O, ΔCO_2_, and carbon flux, ensuring data acquisition occurs only once all three parameters have stabilized. While this increases the accuracy and reliability of the collected data, this can be problematic for throughput for typical carbon assimilation curves that involve many different step changes in CO_2_ since each step requires equilibration time that can collectively greatly extend the length of the protocol. Long equilibration times exacerbate the experimental tradeoff between high data resolution (more observations per A/Ci curve) and the practical feasibility of collecting biological replicates (*ie*, fewer observations per curve, with more replicates for statistical analysis).

**Figure 2:**
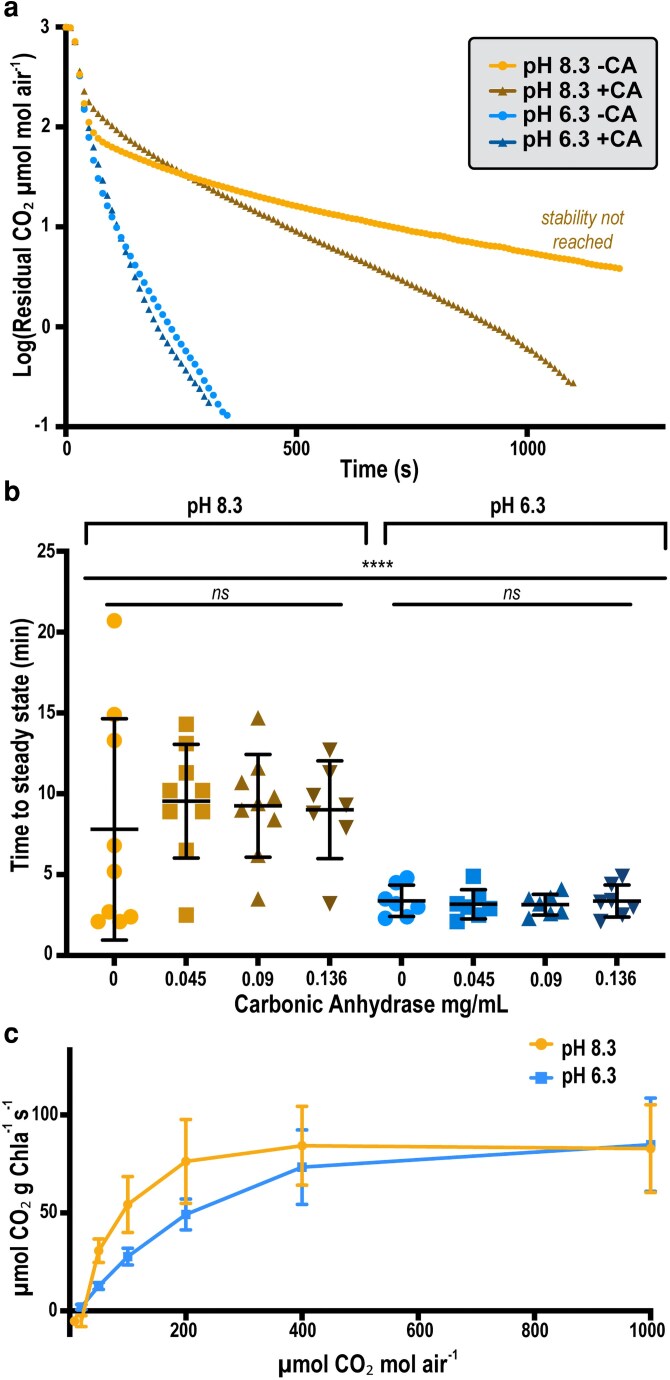
Carbonic anhydrases (CA) and pH influence sampling time duration and variability during LAC CO_2_ assimilation measurements. (a) Residual CO_2_ retained in media during a stepdown change in supplied CO_2_ from 1,000 ppm (*ie*, 0.1%) to 0 ppm in cell-free BG11 media at pH 8.3 or 6.3, with or without exogenously added CA. (b) Time (min) required for a reference sample of cell-free BG11 at pH 8.3 or 6.3 to reach steady-state with increasing concentration of exogenously added CA. (c) CO_2_ assimilation curves of wildtype (WT) cells normalized to Chl*a* in BG11 at pH 8.3 or 6.3. Statistical analysis in (b) shows mean and standard deviation of ≥7 technical replicates, with significance based on ordinary two-way ANOVA (*ns* = no significance, **** = *P* < 0.0001). Error bars in (c) represent standard deviation from the average of 3 technical replicates.

Relatedly, while the alkaline pH routinely used for *S. elongatus* growth (pH ≥8.0) slightly increases CO_2_ solubility it also reduces the efficiency of rapid equilibration between gaseous and dissolved CO_2_. Indeed, when analyzing sterile growth medium (BG11, pH 8.3) as a control, we found the time it took to reach a new steady state following a change in CO_2_ concentration could be highly variable, requiring ∼10 min or more per time point (Fig. 2a, b). Such equilibration times caused our “standard” A/Ci curve using a modest number of sample points (6) to require >90 min to run to completion (Fig. 2a). Throughput could be limiting for sufficient biological replicates at this rate, and the extended time increases the risk of cellular adaptation while in the sample chamber, potentially changing underlying biochemical processes of CO_2_ assimilation and biasing results (Kupriyanova et al. 2023).

We therefore evaluated the impact of pH on sampling rates, due to the well-known relationship of acidity on CO_2_ disproportionation and bicarbonate solubility. Decreasing the pH of the growth medium used for measurements from 8.3 to 6.3 led to an accelerated and less variable equilibration time: from 7.80 ± 6.8 min to 3.38 ± 0.96 min (Fig. 2a, b). This sampling time is comparable to that for plant measurements. We found that, lowering media pH from slightly alkaline (8.3) to slightly acidic (6.3) has no significant effect on the overall assimilation rate or growth rate of *S. elongatus* (Fig. 2c and Figure S1).

We further evaluated the impact of adding CA as an enzymatic catalyst to increase the CO_2_ to HCO_3_^−^ interconversion. CAs are ubiquitous enzymes utilized by cyanobacteria in inorganic carbon scavenging due to their rapid catalysis of the interconversion of CO_2_ and bicarbonate (DiMario et al. 2018). The addition of bovine CA to BG11 medium (*ie*, pH buffered ∼8.3) reduced the variation in time to log a stable steady-state measurement from ±6.84 to around ±3.021 min (Fig. 2b). However, the average length of time required remained similar, or even slightly increased when the medium was supplemented with CA, from 7.80 min at 0 mg/mL to 9.01 min at 0.13 mg/mL (Fig. 2b). Similarly, addition of CA to BG11 pH 6.3 did not significantly reduce average wait time, but diminished variation among sample points from ±0.96 min at 0 mg/mL to ±0.64 min at 0.09 mg/mL (Fig. 2a, b). This result is in general agreement with LI-COR operational instructions to use ≥0.03 mg mL^−1^ CA to hasten time to steady-state (Hupp et al. 2021). In total, pH can be modulated to decrease sampling times, while addition of 0.09 mg/mL CA significantly reduces variation in sampling time: combining these factors reduced the overall time required to measure our standard A/Ci curve by ∼57% (Fig. 2). With these modifications, we were able to achieve a benchmark goal of analyzing 3 different lines of cyanobacteria, in triplicates, within an 8 to 10 hour working day.

### Controlling for artifacts and common instrumentation limitations

**Figure 3:**
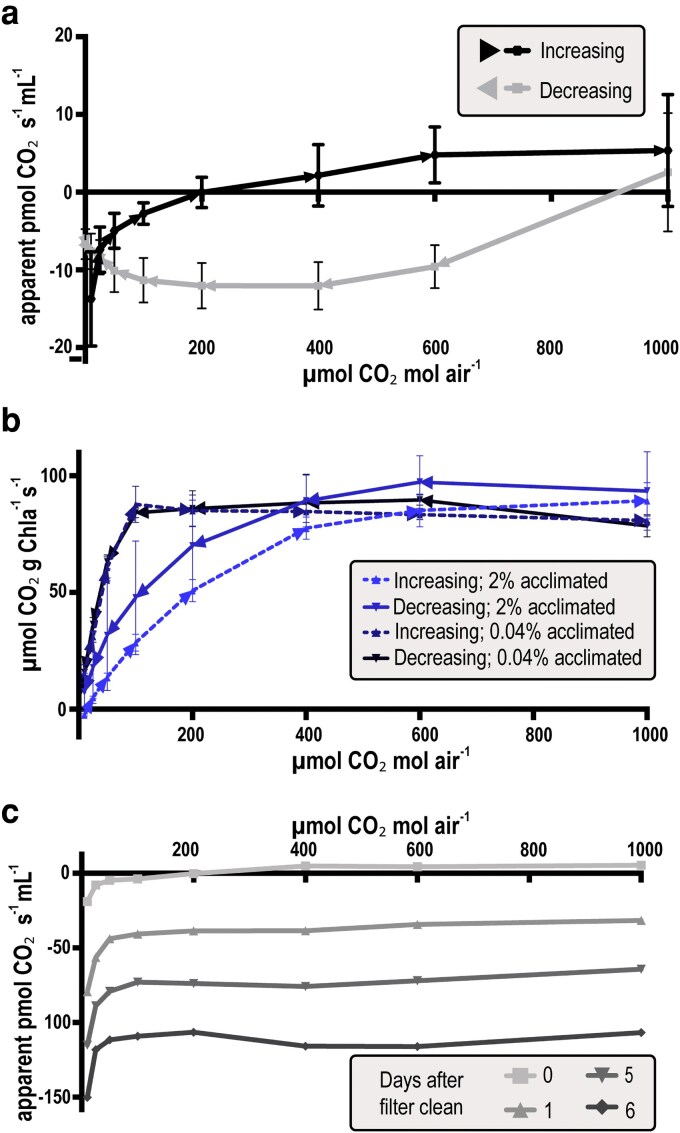
Gas leakage and biofouling affect apparent CO_2_ assimilation measurements. (a) Average and standard deviation of apparent CO_2_ assimilation rates of cell-free BG11 at pH 6.3 starting from 0 to 1,000 ppm CO_2_, (directionality “Increasing”), and from 1,000 (0.1%) to 0 ppm (directionality “Decreasing”), *n* = 7. (b) Average and standard deviation of 3 technical replicates of WT culture adapted to 2% or 0.04% CO_2_ tested with increasing and decreasing CO_2_. (c) CO_2_ assimilation rates of cell-free BG11 pH 6.3 with CA without cleaning the PTFE membrane between different days of measurements.

Initial benchmarking experiments of carbon assimilation curves using WT strains were confounded by differences in CO_2_ directionality and significant variation in between-day replicability. Here, “directionality” refers to the scanning mode used to sample across different CO_2_ concentrations: where responses can be monitored via stepwise increases (low to high CO_2_ ppm) or decreases (high to low) in supplied inorganic carbon. One possible explanation for the apparent differences in measured carbon assimilation from A/Ci curves of different directionality could be technical specifications in the instrumentation, such as insufficient equilibration times. Replication difficulties of A/Ci curves across different-day experiments prompted us to evaluate cell-free chamber background levels of “apparent assimilation” by scanning with increasing or decreasing CO_2_ availability. We observed significant bias in apparent assimilation rates correlated with the difference between the measurement value inside the LAC and ambient CO_2_ (*ie*, 0.042% or ∼420 ppm; Fig. 3a). Further, we observed that this bias behaved differently depending on the direction of ramping of supplied CO_2_ concentration between data points (Fig. 3a). This artifact remained consistent and could therefore be corrected using a standardized curve generated from an axenic chamber control.

Alternatively, biological adaptation of cells while being monitored in the sample chamber could contribute to differences observed in CO_2_ directionality. Cyanobacteria are known to conditionally regulate CCM genes in response to environmental CO_2_ availability, so the duration of an exposure to at or below ambient CO_2_ supply could influence the adaptive state of the suspended cells and influence apparent CO_2_ assimilation rates (Burnap et al. 2015; Desmarais et al. 2019; Sun et al. 2019). To determine if the duration of an A/Ci curve might be sufficient exposure to trigger CO_2_ adaptative responses, we evaluated WT cells adapted to ambient or 2% CO_2_ scanning in either directionality (Fig. 3b). As expected for ambient grown cells (under CO_2_-limiting conditions), no change in apparent assimilation was observed between scanning directionalities for WT cells cultivated under ambient atmospheric CO_2_. However, significant changes in apparent assimilation rates were measured in 2% CO_2_ adapted cells over the time required to obtain an A/Ci curve (Fig. 3b). Most notably, the *A*_max_ (maximal rate of CO_2_ assimilation) is substantially repressed in cultures scanned from low-to-high CO_2_ levels, consistent with prior results shown in *F. diplosiphon* and other cyanobacteria exposed to lower CO_2_ (Rohnke et al. 2020). To minimize the influence of low-CO_2_ exposure on cellular adaptation in subsequent experiments, we therefore use a high-to-low scanning protocol for A/Ci curves and conduct light response curves at 0.1% CO_2_ (1,000 ppm).

Biofouling was another feature of the LAC that frequently led to variability in apparent CO_2_ assimilation rates across different days. The LAC design features a liquid chamber that contains the measured sample and connects to a mixed airflow supplied through water-impermeable PTFE membranes. In our hands, these membranes were prone to biofouling, which we found could substantially influence apparent carbon assimilation rates if not monitored and corrected via background subtraction (Fig. 3c). We found heterotrophic microbial contaminants tended to accumulate on the PTFE membranes over time, presumably promoted by organic matter originating in the sampling chamber. Practically, this contamination increased the level of background respiration to levels comparable to or exceeding that of the carbon fixing by cyanobacteria loaded in the chamber and requires manual cleaning before measurements (Fig. 3c). While background artifacts related to physical features of the gas chamber (*eg*, gas leaks) can be adequately controlled, microbial contamination is more difficult to correct as the respiration level caused by biological activity can vary considerably over relatively short periods. Further, the background respiration caused by biofouling varies throughout a given day, likely caused in part by wetting of the PTFE membrane and subsequent resumption of contaminant metabolic activity.

### Gas exchange reveals distinct classes of carboxysome shell protein mutants

**Figure 4:**
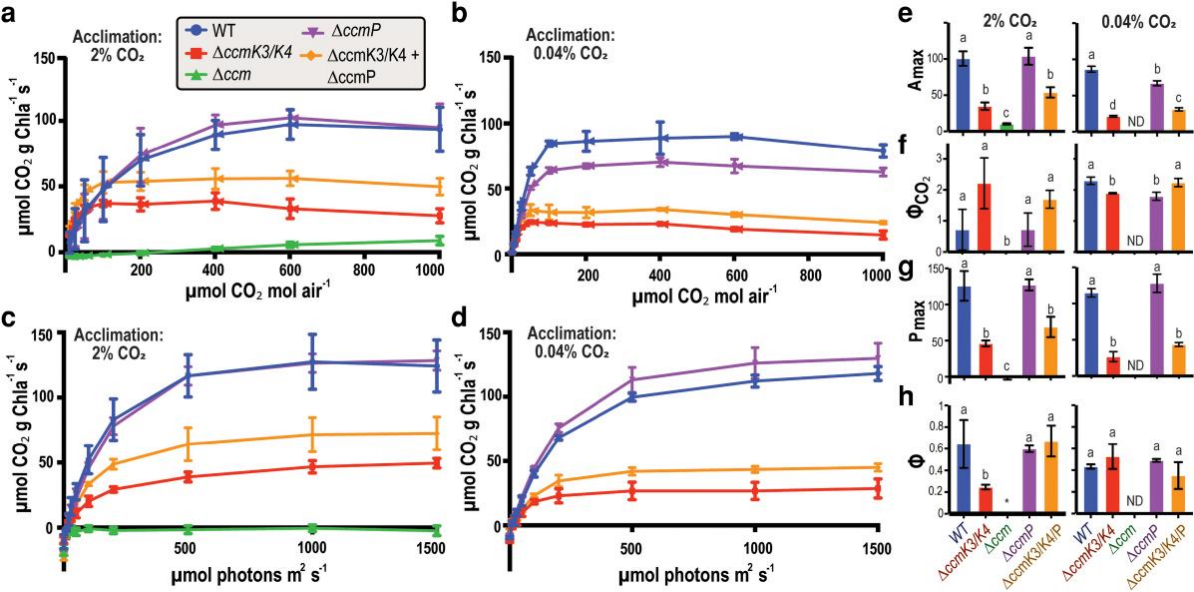
Gas exchange rates reveal an “underappreciated” role for minor shell proteins in cyanobacterial CCM. (a) A/Ci curves of the indicated *Synechoccocus elongatus* cultures acclimated to growth under 2% CO_2_ or (b) ambient air headspace. (c) Light response curve at 0.1% CO_2_ (1,000 ppm) of cultures acclimated to growth under 2% CO_2_ or (d) ambient air. (e–h) Photosynthetic parameters calculated from the assimilation curves in (a–d); include (e) maximum rate of CO_2_ assimilation (*A*_max_); (f) CO_2_ efficiency of assimilation (Φ_CO2_); (g) maximum photosynthetic rate (*P*_max_); and (h) maximum quantum efficiency (Φ). Values from E-H are shown for the indicated cultures grown under 2% (left) or ambient (right) CO_2_ levels. ND = not determined (due to lethal phenotype of Δ*ccm* under ambient CO_2_). All error bars represent standard deviation from *n* = 3 replicates. Different letters in E-H indicate statistical significance *P* < 0.0001 between groups as determined by one-way ANOVA.

We next sought to use the optimized protocols to evaluate the capacity of the system to discriminate photosynthetic performance of cyanobacterial carboxysome mutants. As a reference strain, we generated a Δ*ccmK2LMNO* line (Δ*ccm*), which has been previously reported to lack carboxysomes and exhibit a high CO_2_-requiring phenotype (Friedberg et al. 1989; Price et al. 1993; Cameron et al. 2013) (Figure S2). We wished to compare this severe carboxysome defective mutant to mutants in the peripheral carboxysome shell proteins CcmK3, CcmK4, and CcmP, which functions have not been definitively established. We therefore utilized homologous recombination to generate strains deleted for the coding regions of *ccmP*, the *ccmK3* and *ccmK4* operon (hereafter denoted as the Δ*ccmK3/K4* background*)*, or both genomic loci. The reference Δ*ccm* mutant showed a severe disruption of CO_2_ assimilation under all tested conditions of an A/C_i_ curve, including a ∼10-fold reduction in *A*_max_ compared with WT (Fig. 4a, e). The A/I curve which compares carbon fixation at increasing light intensity, also corroborated severe defects in the Δ*ccm* line, which displayed a maximum photosynthetic rate (*P*_max_) of −1.74 ± 4.32, compared with the WT of 125.73 ± 20.60 µmol CO_2_ g Chla^−1^ s^−1^ (Fig. 4c, g and Table 1). Therefore, the Δ*ccm* strain is incapable of significant net CO_2_ uptake even under 0.1% CO_2_ (1,000 ppm), which is in general agreement with the established high-CO_2_ requiring phenotype of this strain.

**Table 1:** Photosynthetic parameters of cells acclimated to 2% CO_2_. Each value is the average ± standard deviation of 3 replicates.

Variable	Units	WT	Δ*ccmK3*/*K4*	Δ*ccm*	Δ*ccmP*	Δ*ccmK3*/*K4/P*
***A*_max_**	µmol CO_2_ g^−1^ Chla s^−1^	100.47 ± 9.95	34.74 ± 5.13	10.23 ± 0.9	103.7 ± 11.67	53.79 ± 7.06
***C*_1/2_**	µmol CO_2_ mol^−1^ air	120.23 ± 77.58	11.08 ± 3.36	431.2 ± 29.84	109.64 ± 61.45	16.09 ± 1.22
**Γ**	µmol CO_2_ mol^−1^ air	3.64 ± 2.74	2.08 ± 0.55	182.22 ± 27.32	7.82 ± 4.5	0.14 ± 0.81
**Φ_CO2_**	none	0.72 ± 0.66	2.21 ± 0.82	0.02 ± 0	0.72 ± 0.53	1.7 ± 0.29
***R*_Light_**	µmol CO_2_ g^−1^ Chla s^−1^	−1.95 ± 0.92	−5.31 ± 2.76	−3.81 ± 0.71	−5.56 ± 1.55	−0.4 ± 1.87
***V*_max_**	µmol CO_2_ g^−1^ Chla s^−1^	124.57 ± 23.71	36.26 ± 5.81	na ± na	128.87 ± 24.66	56.93 ± 7.48
***K*_m_**	µmol mol^−1^	196.11 ± 143.23	10.55 ± 3.86	na ± na	185.64 ± 120.85	15.28 ± 1.91
***P*_max_**	µmol g^−1^ Chla s^−1^	125.73 ± 20.6	45.98 ± 4.23	−1.74 ± 4.32	127.13 ± 7.7	68.92 ± 14.1
***I*_1/2_**	µmol m^−2^ s^−1^	118.92 ± 37.87	111.71 ± 13.29	4.27 ± 1.51	127.83 ± 14.03	77.92 ± 27.54
***I*_sat_**	µmol m^−2^ s^−1^	525.3 ± 163.59	497.57 ± 61.4	na ± na	570.52 ± 66.01	358.6 ± 120.69
***I_c_***	µmol m^−2^ s^−1^	11.37 ± 0.76	14.75 ± 4.46	na ± na	17.96 ± 6.25	21.83 ± 1.96
**Φ**	None	0.64 ± 0.22	0.25 ± 0.02	4.23 ± 1.97	0.6 ± 0.03	0.67 ± 0.14
***R*_dark_**	µmol CO_2_ g^−1^ Chla s^−1^	−9.07 ± 2.6	−4.33 ± 0.95	−17.04 ± 4.32	−12.81 ± 3.46	−15.28 ± 2.04
**—**	pmol CO_2_ foci^−1^ s^−1^	3.76e-7 ± 3.67e-8	3.26e-7 ± 6.26e-8	1.89e-7 ± 1.2e-8	5.38e-7 ± 8.74e-8	4.32e-7 ± 7.27e-8

It is established that environmental conditions, including CO_2_ concentration, influence expression of CCM genes, including factors such as bicarbonate transporter levels (Burnap et al. 2015; Desmarais et al. 2019; Sun et al. 2025). Therefore, we pre-acclimated strains to ambient CO_2_ levels prior to gas exchange analysis to assess the impact of genetic regulation of CCMs in different mutant backgrounds. WT strains acclimated to ambient CO_2_ levels exhibited an approximate ∼20% reduction in *A*_max_ to 86.39 ± 4.08 µmol CO_2_ g Chla^−1^ s^−1^ (Fig. 4b, e, Table 2). Consistent with an upregulation of CCM factors induced by ambient CO_2_, the CO_2_ concentration required by WT strains to reach half *A*_max_ (*C*_1/2_) was greatly reduced, from 120.23 ± 77.58 to 21.36 ± 1.56 µmol CO_2_ mol air^−1^ (Fig. 4a, b, Table 1, Table 2). The apparent CO_2_ efficiency (Φ_CO2_) was significantly enhanced in WT strains grown in ambient CO_2_ levels 2.29 ± 0.12 compared with 0.72 ± 0.66 at 2% CO_2_-adapted, while *P*_max_ was unchanged between adapted states, consistent with adaptive changes limited to carbon transport and further supported by stable chlorophyll concentration (Fig. 4d, f, g and Figure S3). As expected, the Δ*ccm* mutant was unable to grow under ambient atmosphere (Figure S2), preventing analysis of acclimation to lower CO_2_ in this strain.

**Table 2:** Photosynthetic parameters of cells acclimated to ambient air. Each value is the average ± standard deviation of 3 replicates.

Variable	Units	WT	*ΔccmK3/K4*	Δ*ccm*	Δ*ccmP*	*ΔccmK3/K4/P*
***A*_max_**	µmol CO_2_ g^−1^ Chla s^−1^	86.39 ± 4.08	21.39 ± 1.13	na ± na	67 ± 3.4	31.03 ± 2.13
***C*_1/2_**	µmol CO_2_ mol^−1^ air	21.36 ± 1.56	6.94 ± 0.09	na ± na	20.94 ± 2.49	8.53 ± 0.49
**Γ**	µmol CO_2_ mol^−1^ air	1.96 ± 0.25	1.09 ± 0.24	na ± na	1.69 ± 0.06	1.34 ± 0.2
**Φ_CO2_**	None	2.29 ± 0.12	1.89 ± 0.02	na ± na	1.8 ± 0.13	2.24 ± 0.13
***R*_Light_**	µmol CO_2_ g^−1^ Chla s^−1^	−5.67 ± 0.53	−2.44 ± 0.45	na ± na	−3.86 ± 0.13	−3.56 ± 0.62
***V*_max_**	µmol CO_2_ g^−1^ Chla s^−1^	93.52 ± 3.97	22.13 ± 1.21	na ± na	72.48 ± 4.06	32.54 ± 1.92
***K*_m_**	µmol mol^−1^	26.15 ± 1.62	7.02 ± 0.2	na ± na	25.28 ± 3.29	9.65 ± 0.26
***P*_max_**	µmol g^−1^ Chla s^−1^	114.83 ± 5.81	26.77 ± 6.9	na ± na	127.97 ± 12.64	43.55 ± 2.64
***I*_1/2_**	µmol m^−2^ s^−1^	148.13 ± 16.57	45.12 ± 17.76	na ± na	145.77 ± 14.87	80.61 ± 14.94
***I*_sat_**	µmol m^−2^ s^−1^	652.14 ± 74.88	213.63 ± 79.88	na ± na	641.56 ± 63.96	359.36 ± 53.92
***I_c_***	µmol m^−2^ s^−1^	11.86 ± 3.3	18.67 ± 4.87	na ± na	11.64 ± 0.98	10.96 ± 10.71
**Φ**	none	0.43 ± 0.02	0.53 ± 0.12	na ± na	0.49 ± 0.01	0.35 ± 0.12
***R*_dark_**	µmol CO_2_ g^−1^ Chla s^−1^	−6.5 ± 1.48	−9 ± 1.12	na ± na	−7.29 ± 0.72	−5.19 ± 4.88

Deletion of the accessory shell protein genes *ccmK3/K4* or *ccmP* resulted in intermediate photosynthetic deficiencies that were context-dependent on environmental conditions. For example, Δ*ccmP* mutants acclimated to the elevated CO_2_ we routinely use for cyanobacterial cultivation (2% CO_2_) was indistinguishable from WT across most photosynthetic parameters (Fig. 4a, b, Table 1). However, when Δ*ccmP* mutants were cultivated under ambient CO_2_ levels, a significant drop in *A*_max_ of ∼20% was observed relative to the WT reference (Fig. 4b, e, Table 2). The photosynthetic performance of Δ*ccmK3*/*K4* mutants was significantly suppressed relative to WT under most conditions, indicative of chronic impairment of carboxysome function (Fig. 4). When acclimated to either 2% or ambient CO_2_, Δ*ccmK3*/*K4* mutants displayed a ∼3- and ∼4-fold reduction in *A*_max_, respectively (Fig. 4e). Furthermore, the maximal rate of photosynthesis also declined by 65 to 75% when acclimated to 2% or ambient CO_2_, respectively (Fig. 4g, Tables 1 and 2; *P*_max_). Finally, Δ*ccmK3*/*K4* strains exhibit a low *C*_1/2_ point and more rapid rise in assimilation rate at very low CO_2_ levels, potentially indicative of a chronic upregulation of CCM adaptations even when grown under 2% CO_2_ headspace (Tables 1 and 2). These results are further supported by a higher Φ_CO2_ and a decrease in the concentration at which the rate of photosynthesis and respiration are equal (Γ) compared with WT (Fig. 4f and Table 1). Curiously, rather than display additive effects, triple knockout mutants Δ*ccmK3/K4*+Δ*ccmP* exhibit intermediate phenotypes indicative of a partial rescue of photosynthetic deficiencies relative to the Δ*ccmK3/K4* background. Under all tested conditions, triple mutants display increased *A*_max_ and *P*_max_ compared with Δ*ccmK4/ccmK4*, although triple mutants retain the increase in Φ_CO2_ (Fig. 4, Tables 1 and 2). These results are consistent with increased growth rates of triple mutants relative to comparison strains, especially under 2% CO_2_ (Figure S2).

### Δ*ccmK3/K4* mutants exhibit significant aggregation of carboxysomes

**Figure 5:**
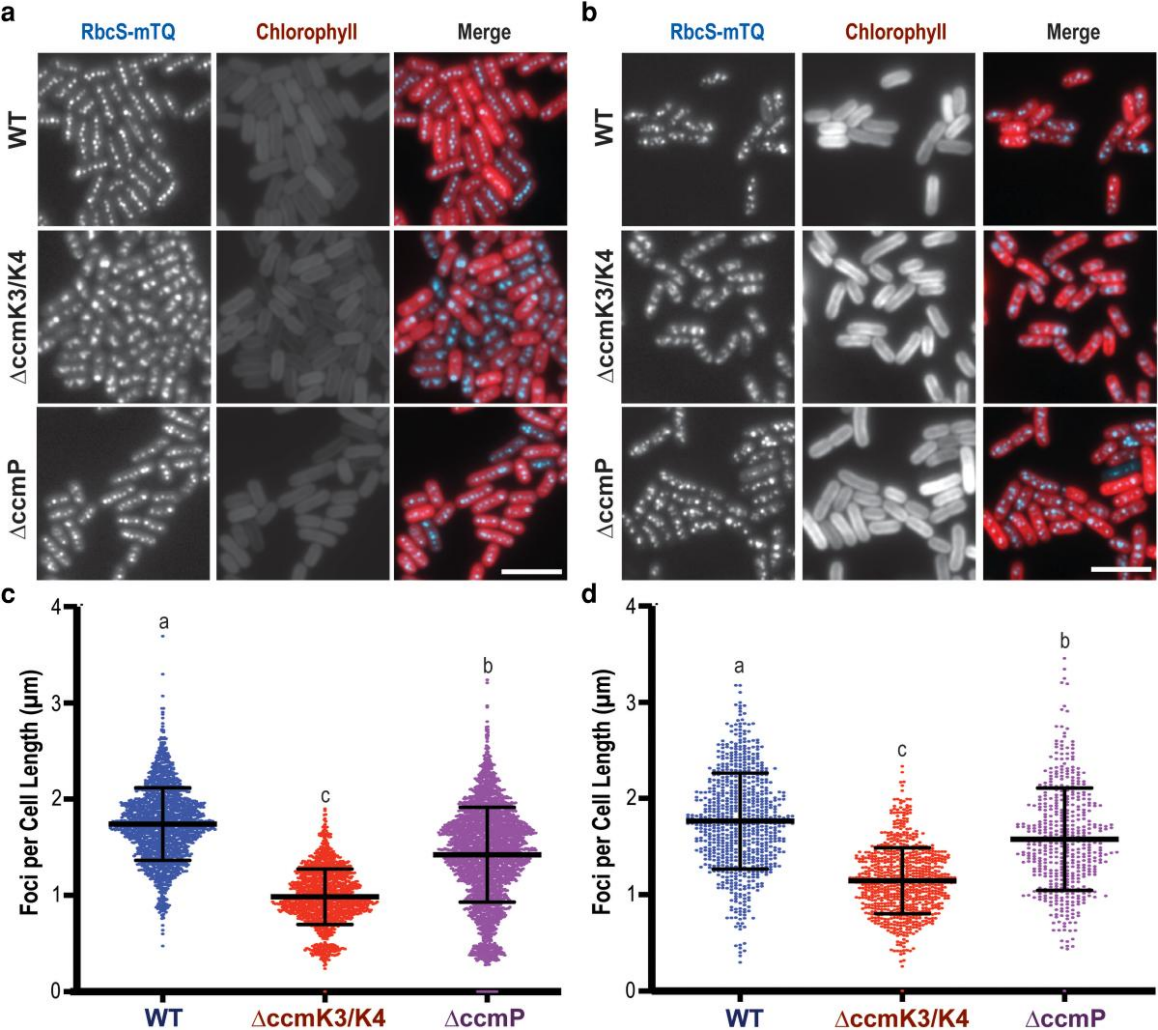
Carboxysome organization and structure in minor shell component mutants. Microscopy of *Synechoccocus elongatus* cells bearing a carboxysome reporter (RbcS-mTQ) reveals carboxysome disorganization, particularly in Δ*ccmK3*/*K4*. Cells are acclimated to (a) 2% CO_2_ or (b) ambient air. Blue = RbcS-mTQ; Red = chlorophyll autofluorescence. Scale bar = 5 µm. The number of carboxysome foci normalized to individual cell length for the indicated strain acclimated to (c) 2% CO_2_ or (d) ambient air. Population average represented in C and D by large, transecting lines, with error bars representing standard deviation. Statistical significates between groups (*P* < 0.0001) indicated by different letters, as assayed by one way ANOVA using *n* ≥ 477 cells per condition.

To correlate assimilation phenotypes with any changes in gross carboxysome number or morphology, we monitored carboxysomes in live cells through the integration of a fluorescent reporter. We integrated an additional copy of the small subunit of rubisco tagged with mTurquoise2 (RbcS-mTQ) that is driven by the endogenous promoter *P*_rbcLS_, a strategy that has been extensively utilized to monitor carboxysome behaviors (Goedhart et al. 2012; Cameron et al. 2013; Maccready et al. 2018; Rillema et al. 2021). We observe that WT cells have increased carboxysome number per cell when grown in ambient air compared with 2% CO_2_, in agreement with previous reports (Whitehead et al. 2014; Sun et al. 2019). However, changing environmental conditions also can lead to cell elongation, and we observe increased average cell length in ambient-grown cultures: when carboxysome number is normalized to cell length (Fig. 5a, b, Figure S4a, b), WT cells maintain an average carboxysome number per cell length of 1.7 ± 0.49 foci/µm regardless of environmental CO_2_ conditions (Fig. 5c, d). Carboxysome number per length is also maintained in Δ*ccmP* mutants across different CO_2_ environments, although a slight decline in overall carboxysome number to 1.5 ± 0.53 foci/µm is observed (Fig. 5). By contrast, Δ*ccmK3*/*K4* cells appear to have fewer carboxysomes per cell length at the resolution limits of light microscopy, although their RbcS-mTQ foci are visibly larger and less well organized in comparison to other strains (Fig. 5c, d). While RbcS-mTQ foci are larger in a Δ*ccmK3*/*K4* background, they are lower brightness in comparison to the average foci in WT, qualitatively indicating a lower density of Rbcs-mTQ within carboxysomes (Figure S5). The increased size of RbcS-mTQ foci in Δ*ccmK3*/*K4* strains could be indicative of larger carboxysome size or of carboxysome aggregation, which has been previously reported in transmission electron microscopy (TEM) analysis of similar ccmK3/K4 mutants (Rae et al. 2012).

**Figure 6:**
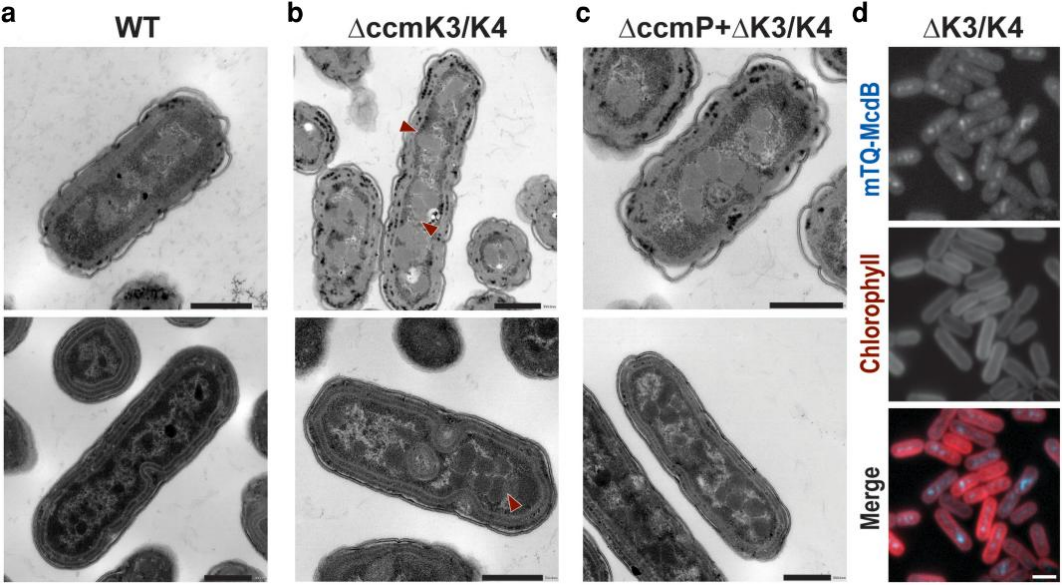
Despite recruitment of positional system components, carboxysome organization is disrupted in Δ*ccmK3/K4* mutants. TEM images of (a) WT, (b) Δ*ccmK3/K4,* and (c) Δ*ccmP*. Arrowheads indicate carboxysomes lacking defined edges typical to these bacterial microcompartments. (d) Representative microscopy images of Δ*ccmK3/K4* strains also bearing a reporter for the carboxysome positional system protein, mTQ-McdB. McdB (blue) remains co-localized to cellular puncta indicative of apparent carboxysome recruitment. Chlorophyll autofluorescence is shown in red. Scale bars in A-C = 500 nm; scale bar in D = 2 µm.

We imaged the Δ*ccmK3*/*K4* background via TEM and verified that the apparent increase in the fluorescence intensity of RbcS-mTQ can be ascribed, at least in part, to aggregation of multiple carboxysomes per foci (Fig. 6b). When normalizing the assimilation rates of different strains, we found that each detectible carboxysome foci in Δ*ccmK3*/*K4* mutants (often representing carboxysome aggregates) had similar assimilation rates to each carboxysome foci in WT background (typically representing a single carboxysome; Table 1 and Figure S6). Additionally, when visualized by both TEM and fluorescence microscopy, a qualitative change in carboxysome integrity was evident in Δ*ccmK3*/*K4* samples. Via TEM, this appeared as presumed aggregates of rubisco that more frequently lacked clear and sharp “edges” indicative of the facets formed by shell proteins (Fig. 6b, arrowheads). In fluorescent reporter backgrounds, the RbcS-mTQ signal in Δ*ccmK3*/*K4* strains appeared to be less focused (Fig. 5a, b), although at the limitations of light diffraction this effect could also be a function of carboxysome aggregation.

The mechanism of carboxysome aggregation in Δ*ccmK3*/*K4* backgrounds remains poorly understood. Possible interpretations could involve improperly formed/separated carboxysomes or a lack of recruitment of cellular machinery required for carboxysome positioning. The carboxysome positioning system consists of one factor that binds to the carboxysome surface McdB (maintenance of carboxysome distribution B) and links the carboxysome to the nucleoid via interactions with McdA (Maccready et al. 2018; Rillema et al. 2021). We therefore analyzed if carboxysome aggregation in Δ*ccmK3*/*K4* is caused in part due to a loss of McdB localization to carboxysomes by tagging the native copy of McdB with mTQ in both a WT and Δ*ccmK3*/*K4* background. Both strains exhibited clear McdB foci co-localized with carboxysome puncta (Fig. 6d), suggesting that carboxysomes in Δ*ccmK3*/*K4* cells can properly recruit elements of the positional system, yet remain aggregated due to other forms of misregulation.

## Discussion

IRGA are a fundamental tool used to measure gas exchange and are widely used to give a range of insights into plant physiology and ecology via assessment of photosynthetic performance, respiration, and stomatal behavior (Long and Bernacchi 2003; Busch et al. 2024). By contrast, CO_2_ gas exchange measurements in aquatic samples are relatively uncommon and require optimization due to CO_2_ solubility limitations and slow equilibrium dynamics. This study highlights how adjusting experimental parameters, such as pH and addition of CA, influences steady-state acquisition and throughput of infrared gas exchange measurements in liquid cultures of the model cyanobacterium, *S. elongatus*, using the commercially-available LI-COR Aquatic Chamber. With suitable adjustment of the experimental setup and controls to minimize instrumentation artifacts, we find that infrared gas analyzers provide data consistent with previously published studies that have used different approaches. For example, we find that the CO_2_ compensation point for WT *S. elongatus* is in close agreement with prior gas exchange measurements (Rohnke et al. 2020; Busch et al. 2024). Additionally, our experimentally determined carbon assimilation rates per carboxysome are closely matched to the range predicted by published mathematical models (Mangan et al. 2016). Additionally, Δ*ccmK2LMNO* (Δ*ccm*) strains are highly deficient in CO_2_ assimilation at all tested CO_2_ concentrations and illumination levels tested, consistent with the severe impairment of the CCM in these strains without carboxysomes (Rae et al. 2012; Cameron et al. 2013). While carboxysome deficient mutants (Δ*ccm*) show expected severe defects in CO_2_ assimilation, mutants in the accessory shell proteins (CcmK3, CcmK4 and CcmP) exhibit nuanced, environmentally dependent phenotypes, highlighting the utility of this approach to quantitatively evaluate subtle photosynthetic phenotypes.

While the carboxysome is one of the best characterized examples of a bacterial microcompartment, the function of several conserved shell proteins remains poorly understood. CcmK3 and CcmK4 are minor shell proteins which form heterohexamers and possess distinct pore features. Similarly, CcmP forms a less common “stacked” dimer of trimer configuration that creates a potential pocket between the dimers that has been hypothesized to be involved in transport of phosphorylated metabolites (Cai et al. 2013; Larsson et al. 2017). It is therefore proposed that peripheral shell proteins outside of the core genomic loci may be differentially expressed to modulate permeability properties of the carboxysome shell under different environments (Sommer et al. 2019; Sun et al. 2025). Gas exchange analysis shows that deletion of *ccmK3* and *ccmK4* or *ccmP* result in intermediate photosynthetic performance relative to strains deleted for the core structural components of the carboxysome (Δ*ccm*). Notably, Δ*ccmP* mutants generally mimic WT strains when acclimated to 2% CO_2_ growth conditions, and that more subtle photosynthetic impairments become evident when the mutants have been acclimated to ambient CO_2_. The A/Ci is sensitive enough to detect small changes in CO_2_ assimilation in Δ*ccmP* (Fig. 4, Table 2) that are not always qualitatively evident by other assays, such as growth curves (Figure S2), frequently used to assess fitness of carboxysome mutants (Sommer et al. 2019).

A further illustration of this is seen in the Δ*ccmK3/K4* mutants, which display more severe defects in CO_2_ assimilation rates which are chronic across the conditions we tested. Δ*ccmK3/K4* strains show a decline in the maximal assimilation rate of CO_2_ (*A*_max_) of ∼65 to 75% relative to WT (Fig. 4e). The relatively steep rise in assimilation at the lower [CO_2_] of the A/C_i_ curve (Φ_CO2_) is consistent with the interpretation that these mutants may be locked at a high induction of other components of the CCM (*eg*, expression of bicarbonate transporters) regardless of if environmental levels of inorganic carbon are elevated (Fig. 4). Despite these significant photosynthetic defects, we observe only small, statistically-insignificant decreases in growth rate for Δ*ccmK3/K4* strains (Figure S2), further highlighting the relative insensitivity of growth rate as an indicator of photosynthetic performance. We note that a decrease in growth rate has been previously shown for Δ*ccmK3/K4* mutants, and it is unclear if our results differ due to a different design for the genetic knockout or if variability in growth conditions routinely used for *S. elongatus* cultivation (*eg*, light, CO_2_, temperature) are responsible for the differences we observe here (Sommer et al. 2019). Regardless, growth is a complex phenotype and reliance on planktonic doubling times or colony survival on solid agar may disguise underlying impairments in photosynthesis.

Our data has additional implications for the role of peripheral shell proteins in the functioning of the carboxysome. Significantly, the pleotropic phenotypes of carboxysome aggregation and structural disorganization of Δ*ccmK3/K4* further indicate that these proteins are not functionally redundant with the homologous dominant hexameric protein, CcmK2. The large carboxysomal aggregates we observe by fluorescence microscopy and TEM maintain association with the positional system component McdB (Fig. 6d) and the aggregates themselves are spaced along the cell length. Yet these aggregates correlate to a decrease in assimilation which has been previously observed. It is therefore possible that CcmK3 and/or CcmK4 may play underappreciated structural roles in carboxysome biogenesis that may manifest as incompletely enclosed/separated carboxysomes that retain physical attachments (Rillema et al. 2021). Therefore, it remains unclear to what extent the photosynthetic impairment of Δ*ccmK3/K4* is contributed by clustering (see Rillema et al. 2021), poorly formed carboxysomes, misregulation of other CCM components, or a mixture of all three factors. By contrast, the relatively minor photosynthetic phenotypes we observe in Δ*ccmP* mutants, particularly under high CO_2_ conditions, strongly imply that CcmP cannot be the sole method of transport for rubisco substrates (RuBP) and products (3-PGA) across the carboxysome shell. One possible interpretation is that other shell proteins have higher permeability than previously anticipated (see eg, Sarkar et al. 2024), although the size and charge of these metabolites likely preclude passage through the central pores of shell protein tiles. We note that we cannot exclude the possibility that knockout of CcmP (or CcmK3 and CcmK4) leaves structural gaps or more modest defects in the integrity of carboxysome through which rubisco substrates and products can be exchanged. Taken together our findings and prior observation, suggest that so-called “minor” shell components likely play underappreciated roles in maintaining carboxysome structure and optimizing CCM function, particularly under CO_2_-limiting conditions (Sun et al. 2016; Sutter et al. 2016; Larsson et al. 2017).

These results contribute to a growing body of work emphasizing the dynamic and condition-dependent nature of carboxysome composition (Larsson et al. 2017; Sun et al. 2019; Melnicki et al. 2021; Ochoa and Yeates 2021; Sutter et al. 2021; Sun et al. 2025). As global interest in engineering carboxysomes into other photosynthetic organisms (eg, C_3_ plants) grows, understanding these subtle regulatory components becomes increasingly relevant (Long et al. 2018; Chen et al. 2023). Use of gas exchange techniques and associated A/Ci curve analysis would provide sufficient throughput to explore environmental contexts more thoroughly under which alternative shell proteins may be particularly adaptive for the function of the CCM. The use of gas exchange measurements tailored for aquatic microbes also provides a critical bridge between cyanobacterial physiology and more broadly applied plant biology frameworks.

## Conclusion

Overall, our study underscores the importance of methodology refinements to the utility of infrared gas exchange measurements and highlights the functional significance of minor carboxysome shell proteins in maintaining efficient carbon fixation in cyanobacteria. The translation of well-described gas exchange techniques from plants can be leveraged to gain quantitative insights into the photosynthetic impacts of peripheral elements of the light harvesting and carbon fixation machinery in cyanobacteria and microalgae. These approaches can complement more traditional approaches used to characterize photosynthetic mutants in cyanobacteria, such as growth rate, microscopy, chlorophyll *a* fluorescence kinetics and pigmentation changes. A wider adoption of infrared gas analyzers to quantify processes of cyanobacterial and microalgal photosynthesis will therefore increase the sensitivity of analysis and enable translation of results to plant systems where these techniques are extensively employed.

## Materials and methods

### Strain condition and growth

Cultures of *S. elongatus* strains were grown in a BG11 medium (Sigma-Aldrich, C3061) buffered with 1 g L^–1^ HEPES (*N*-(2-hydroxyethyl)piperazine-*N*′-(2-ethanesulfonic acid)) at either pH 8.3 or 6.3 with NaOH, as indicated in the text. For routine cultivation of cultures, an Infors-Multitron photobioreactor incubator with fluorescent (GRO-LUX) lighting (∼125 μmol photons m^−2^ s^−1^) and were supplemented with 2% CO_2_ was used at 30 °C with orbital shaking at 150 rpm. To increase consistency in between-day experiments, cultures were typically maintained with daily back dilution to an OD_750_ of 0.3. Transformations were performed as described (Clerico et al. 2007). For growth curves and experiments related to ambient CO_2_ levels, *S. elongatus* was cultivated in a customized Innova 4230 (New Brunswick) shaker fitted with an LED light array (∼125 μmol photons m^−2^ s^−1^), orbital shaking at 100 rpm, 30 °C, and no supplemented CO_2_ supply. Cyanobacterial knockout strains and reporter lines were generated by homologous recombination, as described in (Rillema et al. 2021).

### LI-COR 6800-18 aquatic chamber

Prior to analysis in the LI-COR Aquatic Chamber, exponential-phase cultures were centrifuged at 4,000*xg* for 10 min at 30 °C and resuspended in 12 mL of either 2% or ambient air adapted 30 °C BG11 to a final concentration of ∼2 µg Chl*a* mL^−1^. 1 mL of the resuspension was used for both OD_750_ measurement and for pigment extraction and chlorophyll *a* (Chl*a*) analysis. The remaining 11 mL was returned to the incubator until sample was run in the chamber. At the time of assay 0.09 mg mL^−1^ of bovine CA (Sigma-Aldrich 9001-03+0) was added to decrease time to steady state. All experiments were run at 30 °C. The LI-COR stability and matching criteria are: for Flux_chl. GasEx slope limit = 1e^6^ to 1e^5^ with Stdev limit = 0.0001 to 0.00015 over a 30 s period; ΔH2O.Meas2 slope limit = 0.07 to 0.1 with Stdev limit = 0.01 over a 20 s period, and; ΔCO2.Meas2 slope limit = 0.05 to 0.1 with Stdev limit = 0.05 to 0.1 over a 20 s period.

### Pigment quantification

The protocol for Chl*a* and carotenoid quantification was adapted from (Zavrel et al. 2015). Briefly, Chl*a* was extracted from cell pellets of 1 mL of liquid culture centrifuged at 17,000*xg* for 1 min and resuspended in 100% −20 °C methanol. Cells were incubated up to 30 min at 4 °C and lysed cell biomass was pelleted by centrifugation at 17,000 *xg* for 10 min at 4 °C. Absorbance was taken at 470, 652, 665, and 720 nm. Estimation of pigment abundance was calculated via the equations below:


Chlaμg/mL=12.9447(A665−A720)

Carotenoidsμg/mL=[1000(A470−A720)−2.86(Chlaμg/mL)]/221

### Growth rate quantification

Growth rate was determined from three replicates of liquid cultures by measuring change in OD_750_ every hour for 2% CO_2_ conditions and every 2 h for ambient air over the first 10 h and then every 12 h grown in either BG11 pH 8.3 or pH 6.3, as indicated. Growth rate was calculated from the most linear portion within the first 24 of early exponential growth. Growth rate = ΔLn(OD_750_)/Δ*t*.

### Fluorescence microscopy and image analysis

Fluorescence images were taken with a Zeiss Axio Observer D1 microscope (63×1.3NA) with an Axiocam 503 (mono-chrome) camera using light from X-Cite 120Q (Lumen Dynamics, Mississauga, Canada). Chla fluorescent signals were taken using filter set 43 (000000-1114-101): excitation BP 545/25, emission BP 605/70, and beam splitter FT570. Strains containing an extra copy of the small subunit of rubisco (*rbcS*) tagged with monomeric Turquoise 2 (mTQ) under native promoter inserted into neutral site 1 (1053nt into Synpcc7942_2498) was used for carboxysome reporter in all lines (Goedhart et al. 2012). Turquoise fluorescent signals were taken using filter set 47 (000000-1196-682): excitation BP 436/20, emission BP 480/40, and beam splitter FT455. Images were process in Fiji using background subtraction 20-50 with sliding paraboloid (Schindelin et al. 2012). These data were further analyzed using MicrobeJ 5.13p (Ducret et al. 2016). In each cell line, cell length detection was performed using the rod-shaped descriptor and thresholding set to 0.4 μm < area < 7.5, 2 µm < length, 0.6 μm < width range < 2 μm, 0 μm < width variation < 0.2 μm, and 0.15 μm < circularity amplitude < 0.35 μm. Carboxysome detection was performed using the Maxima point function with a tolerance of 200, *Z*-score of 50, and an intensity minimum of 700. Associations such as inside, location, distance and spacing were all used at default setting. Graphs and statistical analyses were generated with Graph Pad Prism 10.4.1.

### Transmission electron microscopy

For primary fixation 2 mL of later stage cultures (OD750 ∼1.5) was pelleted and resuspended in 1 mL glutaraldehyde 2%, paraformaldehyde 2% in PBS, pH 7.4. After primary fixation, samples were washed with 0.1 M phosphate buffer and post-fixed with 1% osmium tetroxide in 0.1 M phosphate buffer, in block stain with 1% uranyl acetate, dehydrated in a gradient series of acetone and infiltrated and embedded in Spurr resin. 70 nm thin sections were obtained with a Enuity Ultramicrotome (Leica Mycrosystems) and post stained with uranyl acetate and lead citrate. Images were taken with JEOL 1400Flash Transmission Electron Microscope (Japan Electron Optics Laboratory, Japan) at an accelerating voltage of 100 kV.

### Photosynthesis parameters

Data logged from the LI-COR aquatic chamber was used to calculate relevant photosynthetic parameters (see Busch et al. 2024). Average and standard deviation were taken from curves were fitted to each biological replicate plotted using headspace calculated CO_2_ concentrations (Steensma et al. 2023). Values and equations are defined below:

*A*_max_ is the maximum rate of CO_2_ assimilation and is calculated as the plateau of an A/Ci curve.
*P*_max_ is the maximum rate of photosynthesis and is calculated as the plateau of a light response curve.
*R*_Light_ is the respiration in the light and is calculated as the y-intercept of the A/Ci curve.
*R*_dark_ is the respiration in the dark and is calculated as the y-intercept of a light response curve.

The rate *k*, *I*_1/2_, and *C*_1/2_ and were calculated from a one phase association nonlinear regression of A/Ci and A/I curve by Graph pad Prism 10.4.1.


$$A=R_{light}+(A_{max}-R_{light})\;*\;(1-e^{-kx})$$

$$P=R_{dark}+(P_{max}-R_{dark})\;*\;(1-e^{-kx})$$

The CO_2_ compensation point (Γ) is the concentration at which respiration and photosynthesis are equal and was calculated using:


$$\Gamma =\frac{Ln\left(1-\frac{R_{light}}{A_{max}}\right)}{k}$$

The light compensation point (*I_c_*) is the amount of light at which respiration and photosynthesis are equal and was calculated using:


$$I_{c}=\frac{Ln\left(1-\frac{R_{dark}}{P_{max}}\right)}{k}$$

*L*_sat_ is defined here as the lowest concentration of CO_2_ to reach 95% of *A*_max_ and was calculated using:


$$L_{sat}=\frac{Ln\left(20-\frac{20*R_{dark}}{P_{max}}\right)}{k}$$

Φ_CO2_ is defined here as CO_2_ efficiency and is calculated as the initial slope of the A/Ci.


$$\phi_{co2}=\frac{C_{1/2}-R_{light}}{\ln\;(2)/k}\;$$

Φ is defined as the maximum quantum efficiency and is calculated as the initial slope of the A/I.


$$\phi =\frac{I_{1/2}-R_{dark}}{\ln\;(2)/k}$$

### Accession numbers

Sequence data from this article can be found in the GenBank/EMBL data libraries under accession numbers Q31RK3 (*ccmK3*), Q31RK2 (*ccmK4*), Q31QW7 (*ccmP*), Q8GJM6 (mcdB) and Q31NB2 (rbcS).

## Supplementary Material

kiag221_Supplementary_Data

## Data Availability

All data are incorporated into the article and its online supplementary material.
