# Supplementary material for: Gas exchange measurements of carboxysome mutants reveal insights into cyanobacterial carbon-concentrating mechanisms
Source: Plant Physiol. 2026 Apr 20;201(1):kiag221. doi: 10.1093/plphys/kiag221 (PMC13181412; doi:10.1093/plphys/kiag221)
Supplement: kiag221_Supplementary_Data [file kiag221_supplementary_data.pdf]

Supplementary Figures:

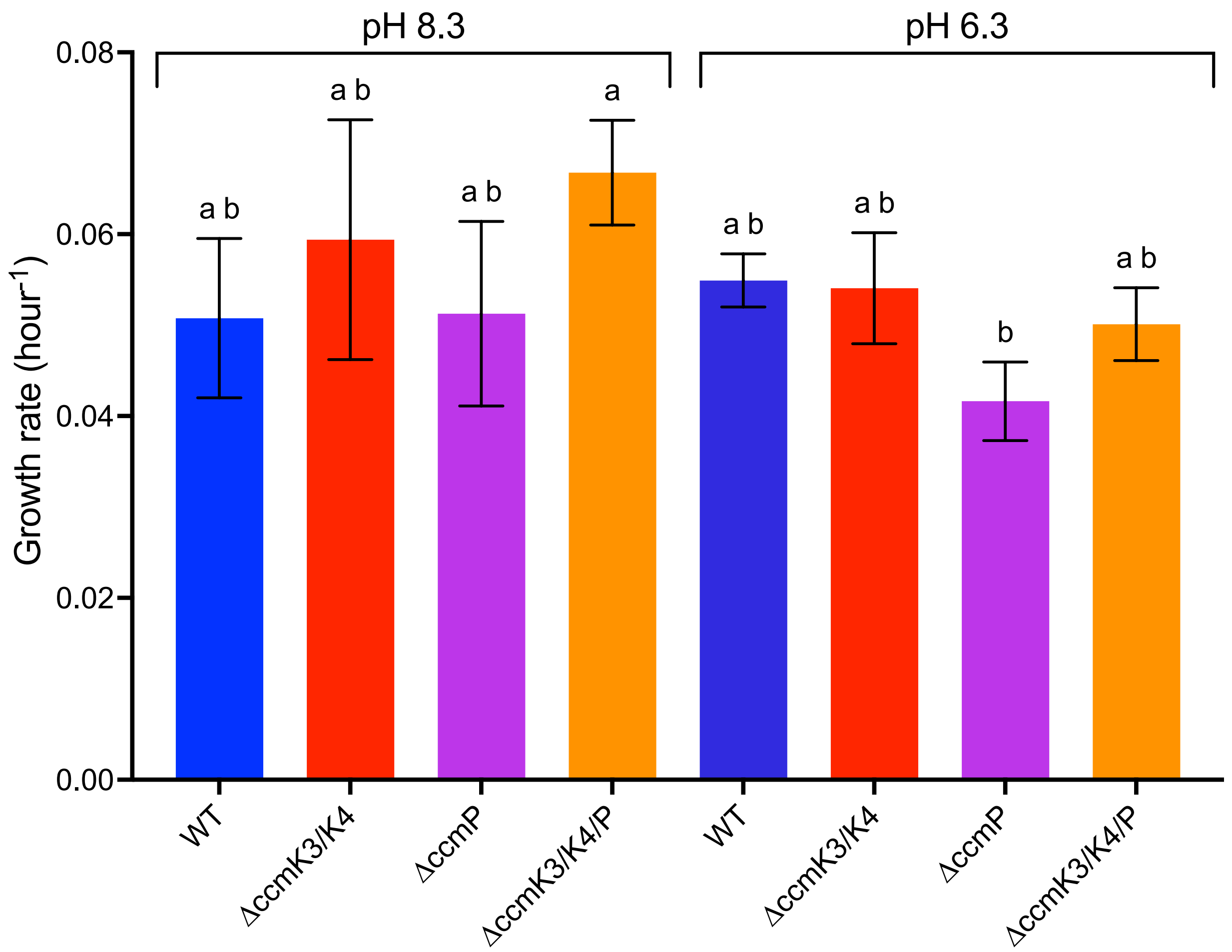

Supplemental Figure S1. Growth rates of cyanobacterial lines used in this study in ambient air with either BG11 pH 8.3 or 6.3. Error bars represent standard deviation from n=3 biological replicates. Letters indicate statistically distinct groups ($p<0.05$) as analyzed by one-way ANOVA.

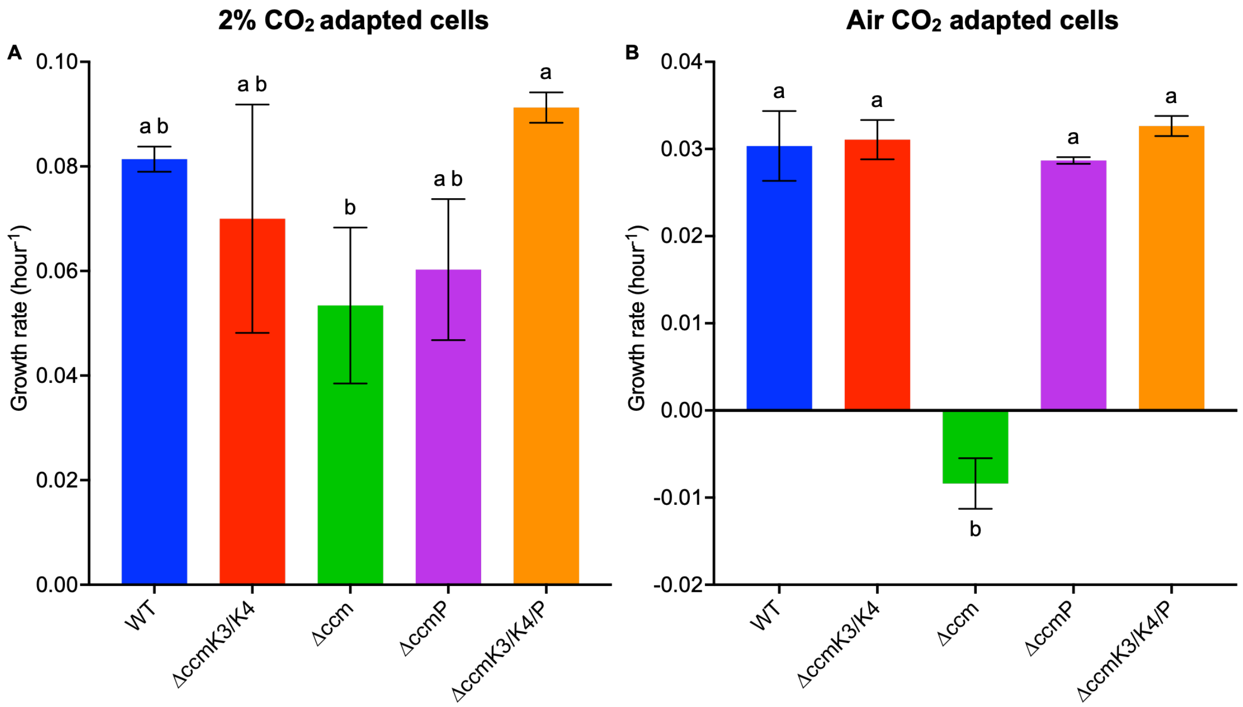

Supplemental Figure S2. Effects of environmental CO_2 on growth rate. **A)** Cells grown at 2% CO_2 **B)** Cells grown at ambient air. Error bars represent standard deviation from n=3 biological replicates. Letters indicate statistically distinct groups ($p<0.05$) as analyzed by one-way ANOVA.

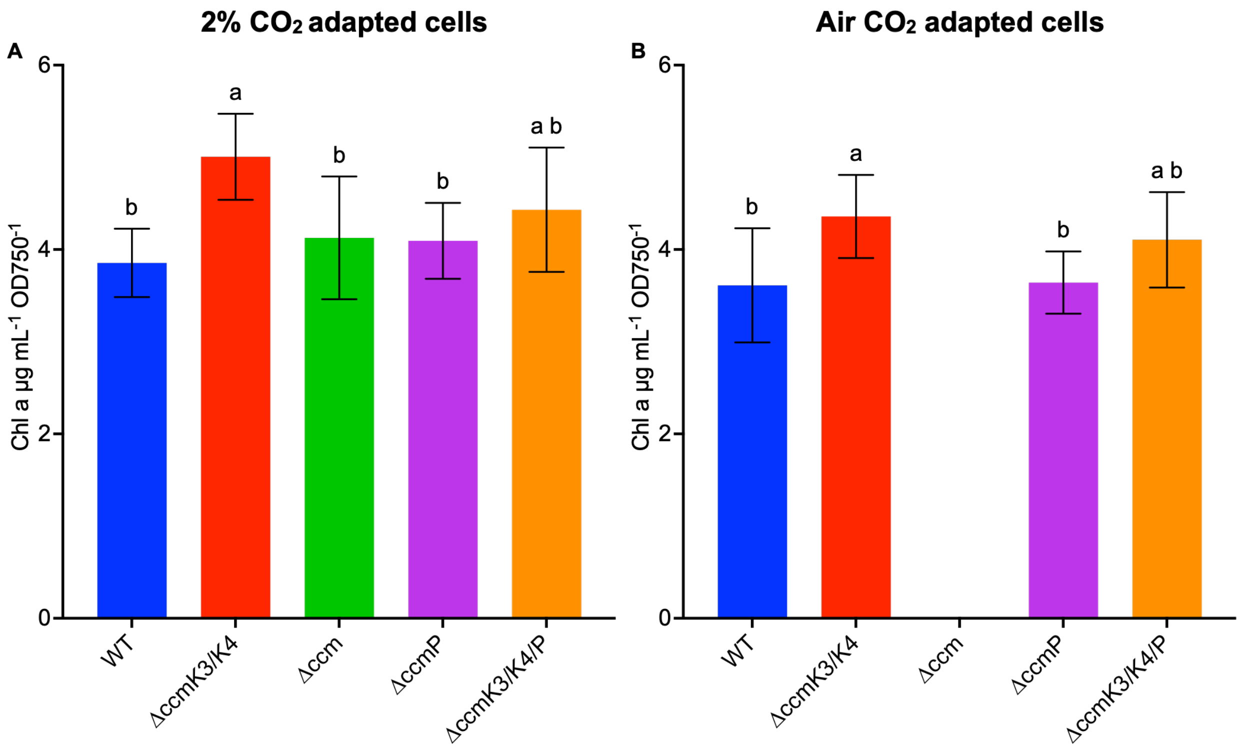

Supplemental Figure S3. Chlorophyll content in carboxysome mutant lines. Changes in Chlorophyll concentrations in cells growth at **A)** 2% or **B)** ambient air. Error bars represent standard deviation from n=3 biological replicates. Letters indicate statistically distinct groups ($p<0.05$) as analyzed by one-way ANOVA.

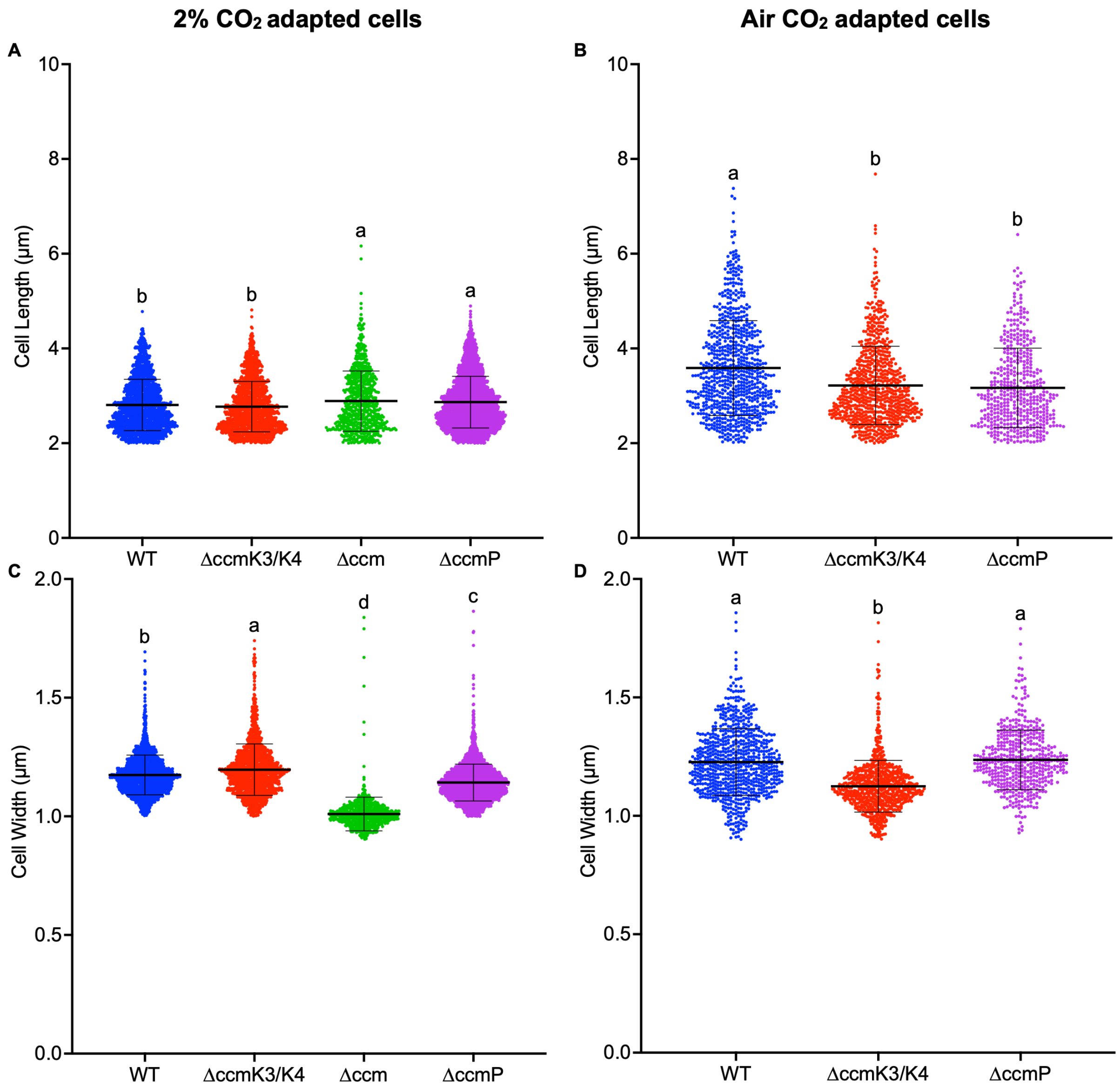

Supplemental Figure S4. Morphological features of carboxysome mutant lines. Cell morphology of **AC)** 2% CO_2 or **BD)** ambient air adapted cells. Large horizontal lines represent averages with error bars denoting standard deviation from $n \geq 477$. Data was collected from microscopy images. Letters indicate statistically distinct groups ($p<0.0001$) as analyzed by one-way ANOVA.

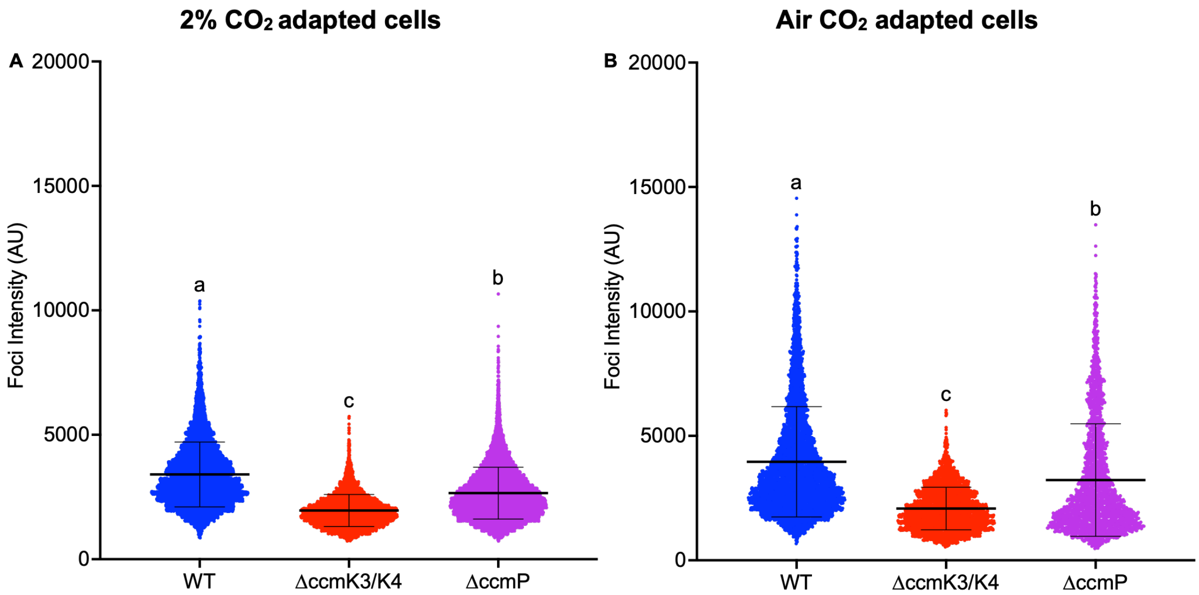

Supplemental Figure S5. Fluorescent intensity of carboxysome foci in carboxysome mutant lines. RbcS-mTQ reporter lines were visualized after growth in **A)** 2% CO_2 or **B)** ambient air adapted cells. Error bars represent standard deviation from n≥2353. Data was collected from microscopy images. Letters indicate statistically distinct groups ($p<0.0001$) as analyzed by one-way ANOVA.

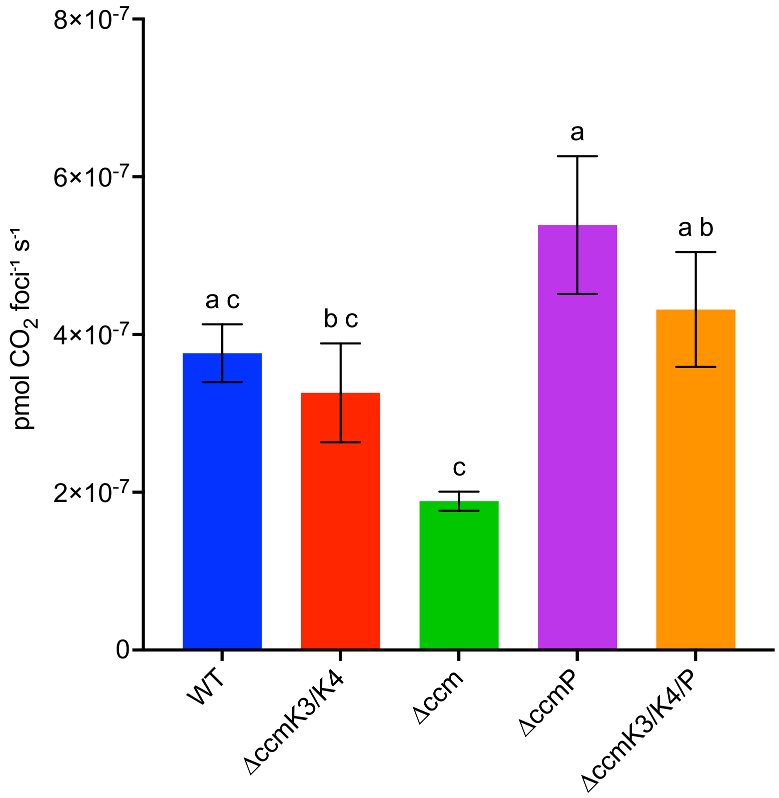

Supplemental Figure S6. Carbon assimilation rates normalized to the average number of RbcS-mTQ foci in the indicated cyanobacterial background strain adapted to 2% CO_2. Assimilation values for Δccm are calculated per cell. Error bars represent standard deviation from n=3. Letters indicate statistically distinct groups ($p<0.05$) as analyzed by one-way ANOVA.
